# Improving health care facility birth rates in Rorya District, Tanzania: a multiple baseline trial

**DOI:** 10.1186/s12884-022-04408-5

**Published:** 2022-01-27

**Authors:** Gail Webber, Bwire Chirangi, Nyamusi Magatti, Ranjeeta Mallick, Monica Taljaard

**Affiliations:** 1 Bruyere Research Institute, University of Ottawa, Ottawa, Canada; 2 Shirati KMT District Hospital, Shirati, Rorya, Mara Tanzania; 3 Ottawa Hospital Research Institute, University of Ottawa, Ottawa, Canada; 4 School of Epidemiology and Public Health, University of Ottawa, Ottawa, Canada

**Keywords:** Facility birth, Community health worker, Birth kits, Misoprostol, M-health, Transportation, Tanzania

## Abstract

**Background:**

Rates of maternal mortality and morbidity in Africa remain unacceptably high, as many women deliver at home, without access to skilled birth attendants and life-saving medications. In rural Tanzania, women face significant barriers accessing health care facilities for their deliveries.

**Methods:**

From January 2017 to February 2019 we conducted a multiple baseline (interrupted time series) trial within the four divisions of Rorya District, Tanzania. We collected baseline data, then sequentially introduced a complex intervention in each of the divisions, in randomized order, over 3 month intervals. We allowed for a 6 month transition period to avoid contamination between the pre- and post-intervention periods. The intervention included using community health workers to educate about safe delivery, distribution of birth kits with misoprostol, and a transport subsidy for women living a distance from the health care facility. The primary outcome was the health facility birth rate, while the secondary outcomes were the rates of antenatal and postpartum care and postpartum hemorrhage. Outcomes were analyzed using fixed effects segmented logistic regression, adjusting for age, marital status, education, and parity. Maternal and baby morbidity/mortality were analyzed descriptively.

**Results:**

We analyzed data from 9565 pregnant women (2634 before and 6913 after the intervention was implemented). Facility births increased from 1892 (71.8%) before to 5895 (85.1%) after implementation of the intervention. After accounting for the secular trend, the intervention was associated with an immediate increase in the odds of facility births (OR = 1.51, 95% CI 1.14 to 2.01, *p* = 0.0045) as well as a small gradual effect (OR = 1.03 per month, 95% CI 1.00 to 1.07, *p* = 0.0633). For the secondary outcomes, there were no statistically significant immediate changes associated with the intervention. Rates of maternal and baby morbidity/mortality were low and similar between the pre- and post-implementation periods.

**Conclusions:**

Access to health care facilities can be improved through implementation of education of the population by community health workers about the importance of a health care facility birth, provision of birth kits with misoprostol to women in late pregnancy, and access to a transport subsidy for delivery for women living at a distance from the health facility.

**Clinical trials registration:**

NCT03024905 19/01/2017.

**Supplementary Information:**

The online version contains supplementary material available at 10.1186/s12884-022-04408-5.

## Background

Despite considerable efforts by governments, international organizations, and civil society stakeholders, maternal deaths in Sub-Saharan Africa remain unacceptably high. Two thirds of global maternal deaths in 2017 were in Sub-Saharan Africa - an estimated 196 thousand women died in this region [1]. Most maternal deaths can be averted by high quality maternal health care by skilled providers in well-equipped settings, however, there are many barriers to care. Thaddeus and Main [2], have described the three delays women experience in accessing health care services at the time of their deliveries: the delay in making the decision to go, the delay in finding transport, and the delay in receiving care once at the health facility. The first delay relates to the understanding of the value of health care services and the weighing of the benefits versus the costs, including cultural preferences. Access to transportation options to access health facilities is a huge challenge for rural residents of lower income countries in Africa and Asia [3] contributing to the second delay. The third delay is related to the quality of health care services; disrespectful and abusive care by health care providers is a common factor in delays in receiving care [4]. Physical equipment and supplies within the facility, the availability of staff to deliver services, and appropriate and timely referrals are also key components [5].

The maternal mortality ratio in Tanzania is at 556 per 100,000 births and has been relatively stable for the last decade. As of 2016, only 50 % of Tanzanian women accessed a health facility at the time of delivery, particularly in the rural northern and western regions of the country [6]. Unfortunately, Tanzania also has the highest rate of perinatal mortality for infants in East Africa [7]. The barriers to receiving health care services for delivery in rural Tanzania can be categorized by the three delay model. Cultural constructs such as a preference for home births and a distrust of the health care system limit decisions to access care [8]. Higher rates of maternal mortality in Tanzania have been associated with lower maternal education and rural residence [9]. Gender norms preventing women having control over the decision to travel and limited transportation options from rural residences to distant health facilities on poorly maintained roads are also well documented barriers [10]. In addition, many of the barriers to care for women are structural, such as limited numbers of skilled health providers, gaps in provision of essential medical supplies, and low health care financing, particularly in the Western and Lake Zones of the country [11]. In our earlier study in Rorya District, barriers to care included distance to health care facilities (ranging from 0.25 km to 57 km for the local health dispensary and up to 90 km for hospital) as well as lack of access and funds for transportation [12].

In order to reduce maternal deaths, ongoing attempts have been made to increase the number of women accessing health facilities at the time of delivery. Punitive strategies, such as fines for home births or withholding health cards for babies born at home, have been shown to be ineffective at encouraging the most marginalized women to access care in rural central Tanzania [13]. In Kasulu District, Kigoma during a discrete choice trial, women have shown a preference for attending health care facilities where providers show respectful attitudes, and there are sufficient medications and equipment [10]. Prior to initiating our trial in Rorya District, Mara Region, we surveyed community members and policy makers about their perceived priorities in improving health care services for women at delivery. The consensus priorities amongst the participants were transportation for women to get to the health facility, the availability of supplies in the health facilities, the number of skilled healthcare providers and improving the healthcare provider attitudes towards women [14]. In preparation of the health care providers for our trial, the negative attitudes of the health care provider towards women were successfully addressed through experiential workshops with nurses using the “Health Workers for Change” curriculum [15].

The aim of this study was to determine the impact of a complex multicomponent intervention on the health facility birth rate (primary outcome) and the rate of antenatal and postpartum care at the health care facilities and postpartum hemorrhage (secondary outcomes) through a multiple baseline trial. The multiple baseline trial was designed to address the barriers of lack of supplies and transport to the health facility, as well as education of the population about the importance of being attended by a skilled health provider in a health care facility.

## Methods

### Study setting

**Fig. 1:**
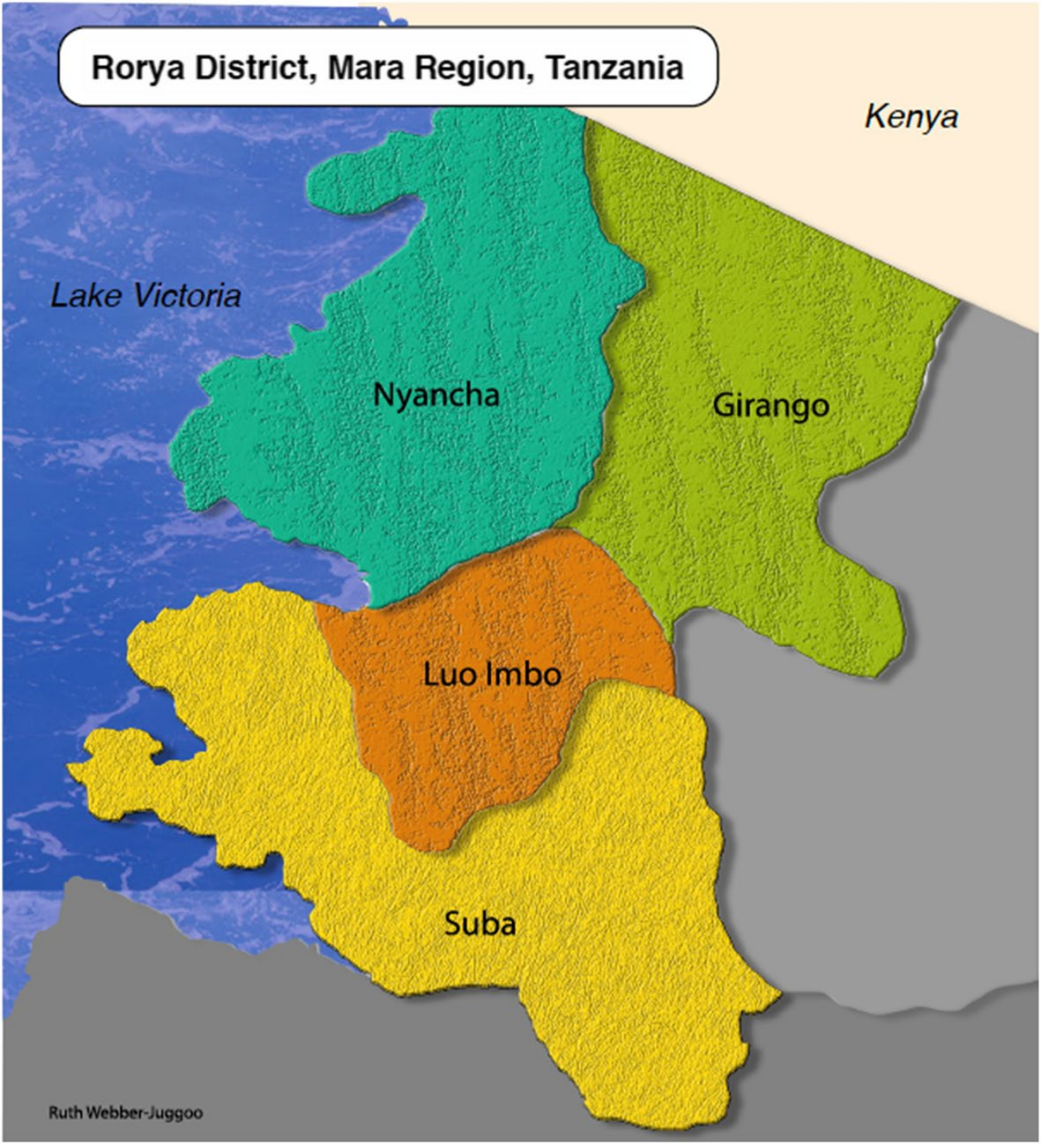
Map of Study Area

The trial was conducted from January 2017 to Feb 2019 in Rorya District of Mara Region, bordered to the west by Lake Victoria, the north by the Kenyan border and the east by Serengeti Park. Geographically, Rorya District consists of four divisions: Nyancha, Girango, Suba and Luoimbo (Fig. 1: Map of Study Area). The study was conducted in all 86 villages of Rorya District.

### Study design

**Fig. 2:**
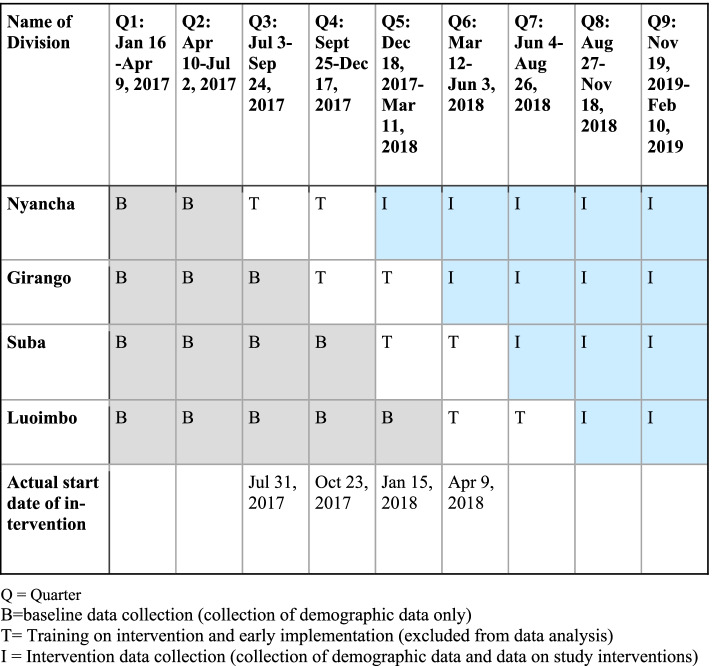
Baseline, Training and Intervention Phases in Four Divisions of Rorya District. Q = Quarter. B = baseline data collection (collection of demographic data only). T = Training on intervention and early implementation (excluded from data analysis). I = Intervention data collection (collection of demographic data and data on study interventions)

We used a multiple baseline interrupted time series design (see Fig. 2 for a study design diagram) to study the impact of the intervention on facility birth rates, antenatal care, and postpartum care. During the baseline period, community health workers (CHWs) registered pregnant women from their villages using mobile phone applications and collected demographic information. Women were exposed to usual care. This was followed by the sequential introduction of the complex intervention in each of the four divisions. The multiple baseline design is a quasi-experimental design which can be used to determine the impact of a complex intervention introduced at a specific point in time [16]. It is superior to many other quasi-experimental and observational designs, such as before and after designs, in that it avoids threats to internal validity such as history and maturation. The basic analytic approach is to conduct a segmented regression analysis of the outcomes in each division of the district. Our design allows us to examine the presence of both an immediate (change in intercept) and gradual effect (change in slope) associated with the intervention.

### Usual care

In this district, women live in isolated villages which may be several kilometres from the nearest health facility (0.25 to 57 km from the local dispensary for women participating in our earlier research [13]). Women are expected to attend the health care facility for antenatal care, at the time of their delivery, and for postpartum visits. As vehicle ownership is non-existent, they usually walk or are reliant on local motorcycle taxis to access the health facility. If the facility lacks medical supplies such as gloves, suture material and medications, they are expected to purchase them from the pharmacy. Historically, many women have chosen not to deliver at the health facility and instead they have relied on traditional birth attendants to deliver them in the villages. This practice of home births has been discouraged by the government as there is evidence that the lack of access to skilled health care providers and appropriate medical supplies results in higher maternal mortality and morbidity [6].

### Interventions

The intervention phase of the research was dependent on the involvement of community health workers and nurses. These were individuals from each of Rorya’s villages and were chosen by community leaders to engage with the women in their villages. They were trained and were provided with a bicycle and a mobile phone to collect the data. The nurses were from local health dispensaries and health centres. One nurse from each of the dispensaries and health centres were trained to participate in the project. During the baseline period, the community health workers recruited the pregnant women and collected demographic information only. During the intervention phase, the complex intervention involved the following activities: firstly, CHWs educated women about the importance of attending the health care facility for antenatal care, delivery and postpartum care and about danger signs in pregnancy (such as headache, visual changes and swelling), using m-health applications on mobile phones. The CHWs also held village meetings to educate the community about the trial and the importance of seeking care at the health care facility. Secondly, nurses and CHWs distributed birth kits to women at 34 to 36 weeks gestation, ideally during their antenatal visits to the nurse, but the CHWs would deliver them to the woman’s home if she did not attend. The birth kits contained clean supplies for delivery such as soap, a delivery mat, gloves, menstrual pad, cord clamps and razor to cut the cord, and 600 mcg of misoprostol to take after delivery as prevention for postpartum hemorrhage. Misoprostol has been used in many settings where access to health facilities is limited [17]. Finally, women living more than two km from the health facility were provided a stipend for the cost of transport to the health care facility at the time of their delivery.

### Primary and secondary outcomes

The primary outcome of the study was health care facility delivery. The secondary outcomes were antenatal care (dichotomized as at least four visits to health care provider during the pregnancy), postpartum hemorrhage (defined as soaking two kangas – a large cloth – in the first day after delivery, a measure of approximately 500 ml [18]) and postpartum care (at least two visits to a health care provider in the first month after delivery). We also recorded deaths and self-reported illness of the mother and newborn (participants and their family members were asked to report deaths or “sickness” in mothers or babies), however, these were not analyzed for trends due to the small numbers.

### Participant eligibility criteria and sample size

No formal power calculation was carried out, but all pregnant women living and delivering in the 86 villages in Rorya District during the period of the trial were eligible to participate. We planned to include monthly data from all pregnant women living in the 4 divisions over 24 months. We anticipated that each division would have approximately 180 births per month for an overall total of over 17,000 births, with a minimum of 6 monthly intervals before and after the implementation of the intervention which was deemed adequate to allow segmented regression analyses to be carried out.

### Delivery of the intervention

We trained the CHWs to use mobile phones to register the pregnant women and collect baseline data in late 2016. We officially began the baseline data collection over the four divisions in January 2017. Initially, we planned to randomize sites to the timing of implementing the intervention, similar to a stepped wedge cluster randomized trial. Randomization of the sites was conducted at a policy maker meeting. The choice of order of divisions to begin the interventions was randomly drawn by one of the policy makers; however, by group decision the order was reversed to start with the more populated divisions. Figure 2 illustrates the schedule implemented for starting the baseline data collection followed by the implementation of the interventions. Note that following the baseline data collection was a period of 6 months of training where the interventions were introduced, however, data collected during these transition periods were not included in the analysis as this training period was used to fully implement the interventions. The actual start date of the interventions in the divisions (listed in the final row) varied during the training periods based on the logistics of organizing training.

### Data collection methods, data management and monitoring

The data were collected by CHWs using mobile phone applications. The women provided the data directly to the CHW except in cases of maternal death, when the family provided the information. It should be noted that the recorded data were all self-reported (including the categories of maternal and infant illness or death). The research team supported the CHWs by regular visits and phone contact to ensure they were using the phone applications appropriately and continuing to collect data. In order to ensure accuracy of the m-health reports, at the end of the project, the data were reviewed with each CHW and cross checked with their written records, then entered into an Excel spreadsheet by the research team.

### Statistical analysis

We compared characteristics of women recruited in the baseline period and the intervention period using descriptive statistics and two-sample t-tests and chi-squared tests. To assess the effects of the intervention, our primary analytical approach was a segmented regression analysis of data across all the sites [19]. The unit of analysis was the individual woman. For the primary outcome, the dependent variable was a binary indicator for facility birth and the model was logistic regression. To account for the secular trend, and to examine immediate and gradual effects of the intervention, we included time (in months) as a continuous variable, an indicator for the condition the division was in each month (baseline control or intervention), and time after the intervention was introduced. The coefficient for the time variable represented the secular trend, the coefficient for the control or intervention indicator represented the immediate effect of the intervention, while the coefficient for the time after intervention variable represented the gradual effect (change in slope). To allow time for the intervention to be fully implemented and for women recruited in the control period to deliver their babies without contamination from the intervention, we censored the first 6 months after implementation from the analysis. To account for potential differences in characteristics of women recruited over time, our analysis adjusted for the following covariates: age (continuous), marital status, and education as well as parity (continuous). Marital status was collapsed by grouping married and partner versus single versus other (widowed, separated or divorced); education was collapsed by grouping none, primary versus other. Age was initially modelled using a restricted cubic spline with three knots; however, model fit statistics (AICC) indicated that a simple linear term for age was adequate. Our primary analytical approach treated the division as a fixed effect. A secondary analysis included the interactions with each of the time, intervention and time after intervention variables to allow the secular trend as well as the immediate and gradual effects of the intervention to vary across the divisions [20]. The estimates from the model were expressed as adjusted odds ratios with 95% confidence intervals (CIs).

The analysis of the secondary outcomes (antenatal care of at least four visits, postpartum hemorrhage, and two postpartum visits in the first postpartum month) were conducted as described for the primary outcome. The maternal and newborn mortality and morbidity outcomes were not analyzed using segmented logistic regression due to very low frequency of events; rather, they were reported descriptively in the before and after periods.

### Ethics

Ethical approval for this study was obtained from the Ottawa Hospital Science Network Research Ethics Board Protocol number 20160815-01H, Bruyere Research Ethics Board (Ottawa) protocol number M16–16-050, and Tanzania’s National Institute for Medical Research Ethics Board protocol number NIMR/HQ/R.8a/Vol. IX/2023. Informed consent was obtained from all the study participants. All methods were performed in accordance with the relevant guidelines and regulations (Declaration of Helsinki). All participants were provided with a consent form which they could read or have read to them and they were asked to sign it if they agreed to participate in the study. A small number of women who participated in both the baseline and intervention phases were asked to sign a new consent form at the start of the intervention phase. All participants were assured that their identity would remain confidential as all data were de-identified and referred to by a numerical code only.

## Results

In total, 12,359 women were recruited to be part of the trial across all four divisions; after eliminating the implementation (training) period, a total of 9565 women contributed to the analysis. The demographic descriptions of these women are presented in Table 1.

**Table 1:** Demographic Characteristics of Enrolled Women

Characteristic	Total (***N*** = 9565)	Baseline (***N*** = 2634)	Post-Intervention (***N*** = 6931)	***P***-value
Age, mean (SD) Range	26 (7) (11–51)	27 (7) (12–48)	26 (7) (11–51)	< 0.0001
Number of previous Pregnancies, median (range)	2 (0–14)	3 (0–14)	2 (0–11)	0.0006
**Marital Status, N(%)**				0.0529
Married	8515 (89.0%)	2368 (89.9%)	6147 (88.7%)	
Single	767 (8.0%)	198 (7.5%)	569 (8.2%)	
Widow	160 (1.7%)	40 (1.5%)	120 (1.7%)	
Separated	55 (0.6%)	19 (0.7%)	36 (0.5%)	
Partner	60 (0.6%)	7 (0.3%)	53 (0.8%)	
Divorced	8 (0.1%)	2 (0.1%)	6 (0.1%)	
**Education, N (%):**				< 0.0001
None	435 (4.5%)	123 (4.7%)	312 (4.5%)	
Primary	8913 (93.2%)	2371 (90.0%)	6542 (94.4%)	
Secondary 4 (4 years of secondary school)	141 (1.5%)	124 (4.7%)	17 (0.2%)	
Secondary complete (6 years of secondary school)	42 (0.4%)	7 (0.3%)	35 (0.5%)	
University	34 (0.4%)	9 (0.3%)	25 (0.4%)	

The mean age of the participating women was 26.5 years and was similar across all divisions (range of means from 26.1 to 26.8 years) and in the before and after periods. The median number of previous pregnancies was 3 in the before period versus 2 in the after period. The majority of the women reported being married (89%), while 8% were single, 1.7% widowed, 0.6% separated, 0.6% living with unmarried partner and 0.1% were divorced. Most of the participating women had primary education only (93.2%).

Table 2 presents the raw (observed) proportions for the primary and secondary outcomes from the pre- and post-intervention phases of the project. The observed trends over time are displayed in the Additional file (Figs. S1-S4). Table 3 contains the results from the pooled segmented logistic regression analysis of the primary outcome (facility birth), showing the estimated secular trend, and the immediate and gradual effects of the intervention. The observed facility birth rate overall rose from 1892 (71.8%) before to 5895 (85.1%) after implementation of the intervention. After accounting for the secular trend, the intervention was associated with an immediate increase in the odds of facility births (OR = 1.51, 95% CI 1.14 to 2.01, *p* = 0.0045) as well as a small gradual effect (OR = 1.03 per month, 95% CI 1.00 to 1.07, *p* = 0.0633).

**Table 2:** Raw (observed) primary and secondary outcome prevalence before and after intervention by district and overall

Outcome	Baseline (***n*** = 2634)	Post-Intervention (***n*** = 6931)
**Facility Birth**		
Girango	467 (63.4%)	1316 (79.4%)
Luo Imbo	202 (81.1%)	209 (79.8%)
Nyancha	687 (71.6%)	3477 (88.2%)
Suba	536 (77.9%)	893 (83.5%)
**Overall**	1892 (71.8%)	5895 (85.1%)
**Antenatal Care of at least 4 visits**		
Girango	310 (42.1%)	786 (47.4%)
Luo Imbo	108 (43.4%)	101 (38.6%)
Nyancha	481 (50.1%)	2218 (56.3%)
Suba	328 (47.7%)	601 (56.2%)
**Overall**	1227 (46.6%)	3706 (53.5%)
**Postpartum Hemorrhage**		
Girango	58 (7.9%)	223 (13.4%)
Luo Imbo	17 (6.8%)	10 (3.8%)
Nyancha	116 (12.1%)	363 (9.2%)
Suba	52 (7.6%)	69 (6.4%)
**Overall**	243 (9.2%)	665 (9.6%)
**Postpartum Care**		
Girango	674 (91.4%)	1575 (95.0%)
Luo Imbo	205 (82.3%)	244 (93.1%)
Nyancha	920 (95.8%)	3895 (98.8%)
Suba	670 (97.4%)	1039 (97.2%)
**Overall**	2469 (93.7%)	6753 (97.4%)
**Maternal Mortality/Morbidity**		
Girango	5 (0.7%)	10 (0.6%)
Luo Imbo	0 (0%)	0 (0%)
Nyancha	4 (0.4%)	10 (0.2%)
Suba	0 (0%)	2 (0.2%)
**Overall**	9 (0.3%)	22 (0.3%)
**Baby Mortality/Morbidity**		
Girango	17 (2.3%)	36 (2.2%)
Luo Imbo	3 (1.2%)	0 (0.0%)
Nyancha	9 (0.9%)	44 (1.1%)
Suba	2 (0.3%)	15 (1.4%)
**Overall**	31 (1.2%)	95 (1.4%)

**Table 3:** Results from the primary segmented logistic regression analysis of the primary and secondary outcomes (*n* = 2634 baseline, *n* = 6931 intervention)

Parameter	Odds Ratio (95% Confidence Interval)	***P***-value
**Facility Births (Primary outcome)**^a^		
Baseline intercept		
Girango	0.57 (0.48 to 0.67)	< 0.001
Luo Imbo	0.93 (0.72 to 1.21)	0.5896
Nyancha	1.04 (0.86 to 1.27)	0.6583
Suba	Ref	
Secular trend (change/month before intervention)	1.01 (0.98 to 1.04)	0.4006
Immediate effect (intercept change)	1.51 (1.14 to 2.01)	0.0045
Gradual effect (slope change)	1.03 (1.00 to 1.07)	0.0633
**Antenatal Care of at least 4 visits**^a^		
Baseline intercept		
Girango	0.77 (0.67 to 0.88)	0.001
Luo Imbo	0.64 (0.52 to 0.79)	< 0.001
Nyancha	1.01 (0.86 to 1.18)	0.9191
Suba	Ref	
Secular trend (change/month before intervention)	1.00 (0.98 to 1.02)	0.9618
Immediate effect (intercept change)	1.19 (0.93 to 1.51)	0.1713
Gradual effect (slope change)	1.02 (0.99 to 1.05)	0.2391
**Postpartum care**^a^		
Baseline intercept		
Girango	0.46 (0.32 to 0.65)	< 0.001
Luo Imbo	0.18 (0.12 to 0.28)	< 0.001
Nyancha	1.86 (1.22 to 2.82)	0.0036
Suba	Ref	
Secular trend (change/month before intervention)	1.08 (1.03 to 1.13)	0.0017
Immediate effect (intercept change)	1.07 (0.61 to 1.89)	0.8116
Gradual effect (slope change)	0.92 (0.86 to 0.98)	0.0125
**Postpartum Hemorrhage**^a^		
Baseline intercept		
Girango	1.95 (1.53 to 2.48)	< 0.001
Luo Imbo	0.75 (0.48 to 1.17)	0.2007
Nyancha	1.57 (1.17 to 2.12)	0.0029
Suba	Ref	
Secular trend (change/month before intervention)	1.01 (0.96 to 1.05)	0.7233
Immediate effect (intercept change)	1.04 (0.68 to 1.61)	0.8500
Gradual effect (slope change)	0.97 (0.92 to 1.02)	0.2271

^a^Results from pooled segmented logistic regression analyses; all analyses were adjusted for age, marital status, education, and parity

For the secondary outcome, antenatal care of at least 4 visits, the observed rate overall rose from 1227 (46.6%) to 3706 (53.5%) after implementation of the intervention. After accounting for the secular trend, the intervention was associated with an immediate increase in the odds of antenatal care (OR = 1.19, 95% CI 0.93 to 1.51, *p* = 0.1713), as well as a small gradual effect (OR = 1.02, 95% CI 0.99 to 1.05, *p* = 0.2391) per month, both of which were not statistically significant. For postpartum care, the observed rate overall rose from 2469 (93.7%) to 6753 (97.4%). After accounting for the secular trend, there was no statistically significant immediate change in the odds of postpartum care (OR = 1.07, 95% CI 0.61 to 1.89, *p* = 0.8116) although there was a statistically significant attenuation (OR = 0.92, 95% CI 0.86 to 0.98, *p* = 0.0125) suggesting a gradual decrease to baseline. For post-partum hemorrhage, the observed rate was virtually unchanged from 243 (9.2%) before to 665 (9.6%) after implementation of the intervention with no significant immediate or gradual change associated with the intervention.

The results from the secondary segmented logistic regression analyses of the primary and secondary outcomes accounting for differences across divisions are presented in the additional file (Supplementary Table 1). There were significant differences across the divisions in the immediate and gradual intervention effects for the primary outcome (facility births), but not for antenatal care of at least 4 visits, postnatal care, nor post-partum hemorrhage. The results for the primary analysis show that the immediate improvement in the primary outcome was driven by Girango Division.

## Discussion

There was a positive impact on our primary variable of facility births. Other studies in Africa have shown improvements in facility birth rates with CHW based m-heath interventions. An increase in facility births was demonstrated by a cluster randomized trial of CHWs using smart phone applications in Singida Rural and Iramba Districts of central Tanzania, and it was particularly effective for first-time mothers and those with limited ANC visits [21]. In the neighbouring country of Rwanda, an interrupted time series of a m-health CHW project using Rapid SMS from 2012 to 2016 demonstrated an increased use of maternal and child health services, however, the Rapid SMS system alone was ineffective; training, supervision and equipment were required to demonstrate a positive impact [22]. The need for continuous training and material support of the CHWs (e.g. solar chargers and upgraded phones) was determined from qualitative focus groups with the Rwandan CHWs [23]. In fact, in their review of m-health programs using CHWs, Braun and colleagues determined that while there is significant potential for CHWs to collect data and provide health services using m-health applications, more research needs to be done on scale-up of these projects to measure their impact on program implementation and health policy [24].

While m-health is a useful tool for data collection, monitoring CHW activities, and education of the population, it is not a necessary component for improvement of the population’s health. In a study in Tanzania’s largest city, Dar es Salaam, a CHW home visit program was effective at further increasing the number of facility births in a region where they are already high [25]. Unfortunately, the impact of CHWs may be limited by other barriers to care. CHW engagement of pregnant HIV positive women in Shinyanga Region of Tanzania was not demonstrated to be effective at helping women stay on medications to prevent mother to child transmission of HIV, likely due to barriers such as transport and HIV stigma and lack of fidelity of program implementation [26]. Similarly, in a cluster-randomized trial using CHWs to improve child health in Rufiji, Kilombero and Ulanga Districts of Tanzania, it was noted that inability to maintain stocks of essential medications eliminated any benefit of the CHW program [27]. In our project, maintaining stocks of the birth kits is particularly important for the viability of the project. This will be an ongoing challenge for the government, for if the supply chain is weak, women may not be motivated to make the effort to attend a health facility for antenatal care or delivery. Using CHWs to educate women and provide birth kits is a key factor to engaging women with the formal health care system; if women experience quality improvement in antenatal services, they are more likely to return for their deliveries, even if they previously gave birth at home [28].

There were several limitations to this study. The four divisions had different sizes: Suba and Luoimbo Divisions had smaller populations and fewer villages. Suba and Luoimbo Divisions were also more remote from the research team who was based Shirati town, in Nyancha Division, therefore it was more challenging to supervise this group of CHWs. There was no incentive for the women to get registered during the baseline period of data collection, thus the numbers of women registered during this phase of the research were particularly low in Suba and Luoimbo Divisions, and the period of data collection during the intervention phase was considerably shorter than for Nyancha and Girango Divisions. This may have contributed to the lack of an effect of the interventions in these two divisions. To the extent that women recruited in the baseline period within each community were systematically different than women recruited in the intervention period in that community, our results may be biased. However, we attempted to adjust for potential risks of bias through covariate adjustment. CHWs were key members of the project, however, they work essentially as volunteers in Tanzania. While we provided them with a small stipend, a bicycle and a mobile phone, they also were occupied with their own work (often agricultural) and could not commit to the project full-time. Hampshire and colleagues note that task-shifting roles to CHWs who are receiving little or no compensation is fraught with problems, and their roles health care system should be formalized with accompanying salaries [29]. In this project, some of the villages in Rorya District are very dispersed and challenging for one CHW to cover, even with a bicycle. Thus it is probable that many pregnant women did not get registered for the trial, particularly during baseline data collection period. During the intervention phase, women were more likely to seek out the CHW to get registered as there was considerable motivation to obtain the birth kits, as documented by our earlier research [30].

The lack of impact of the interventions on the secondary outcomes of antenatal and postpartum care, and postpartum hemorrhage may have several explanations. There is a relatively high baseline rate of antenatal visits in Tanzania [6], thus our interventions did not create any measurable impact. Postpartum hemorrhage is a self reported variable (soaking two kanga clothes in one day), and thus may not be reliably reported. Without access to health care providers and other measurement tools such as weighing of soiled clothes, this is the best measure available.

Postpartum care appears to be more associated with urban status and higher education in Tanzania [6], though as noted earlier, our baseline rates of postpartum care were also high in this rural population. The use of the m-health applications presented other challenges. The instability of the network led to multiple repeat submissions of data, and much time was required to clean up the database. If the Tanzanian government wishes to use m-health data collection in the future, the stability of the network and a strong plan for supervision of the CHWs and data management need to be developed. Finally, the transport subsidy was difficult to implement. Eligible women had to locate the nurse at the health facility on their arrival in order to pay their taxi driver and at times there was insufficient funds at the health facility to cover this cost. This was inconvenient for all involved (the women, taxi drivers and nurses) and we would recommend an alternative method for generating and distributing funds for transport in the future such as a community fund. Finally, this trial was not large enough to show a significant difference in maternal and newborn well-being: a considerably longer trial or larger population would be required. Future researchers may wish to explore causes of maternal and newborn morbidity and mortality from the perspective of medical diagnosis (not based solely on self-report) to determine if the complex intervention could have had an impact.

This study demonstrated that facility birth rates can be improved by a complex intervention including CHW-provided education about the importance of health facility births, late third trimester provision of free supplies in birth kits, and transportation subsidies for women living at a distance from the health facility. These interventions were preceded by conversations with community members and policy makers about their perceived priorities in improving health care services for women at delivery, and a training program to improve health care providers’ attitudes towards women at delivery. The community member and policy maker priorities were established as the components of the intervention; different contexts may require adjustment to the intervention depending on the needs of the population.

## Conclusions

This multiple baseline trial demonstrated that a combination of interventions involving CHWs with mobile phones, distribution of birth kits with misoprostol and transport subsidies were successful at increasing women’s attendance for facility birth. The interventions did not show a demonstrable effect on antenatal care, postpartum care, postpartum hemorrhage. The District Medical Office of Rorya has been convinced that the CHW education and birth kit distribution has been successful, and they have begun to include the birth kits in their budget. The transport subsidy was challenging to implement, and alternative strategies for supporting transport for women are being considered such as a community fund. This trial demonstrates that it is possible to have a positive impact on the facility birth rate by improving services (education, supplies and transport) for women in rural communities in Tanzania. Utilizing CHWs and birth kits is a feasible way to ensure women have access to supplies for delivery even if they cannot afford to purchase them, or if they are unable to reach the health facility due to precipitous delivery. In rural settings, where it may be challenging for women to access health facilities, we recommend a program of education, birth kits and ideally some form of transport support to encourage facility delivery.

## Supplementary Information

**Additional file 1.**

**Additional file 2.**

## Data Availability

All data generated or analyzed during this study are included in this published article or can be found in the supplementary files.

## Abbreviations

ANC: Antenatal care
CHW: Community Health Worker
PNC: Postnatal care
PPH: Postpartum hemorrhage
