# Supplementary material for: Improving health care facility birth rates in Rorya District, Tanzania: a multiple baseline trial
Source: BMC Pregnancy Childbirth. 2022 Jan 27;22:74. doi: 10.1186/s12884-022-04408-5 (PMC8793235; doi:10.1186/s12884-022-04408-5)
Supplement: Supplementary file 1 — Additional file 1. [file 12884_2022_4408_MOESM1_ESM.pptx]

## Slide 1
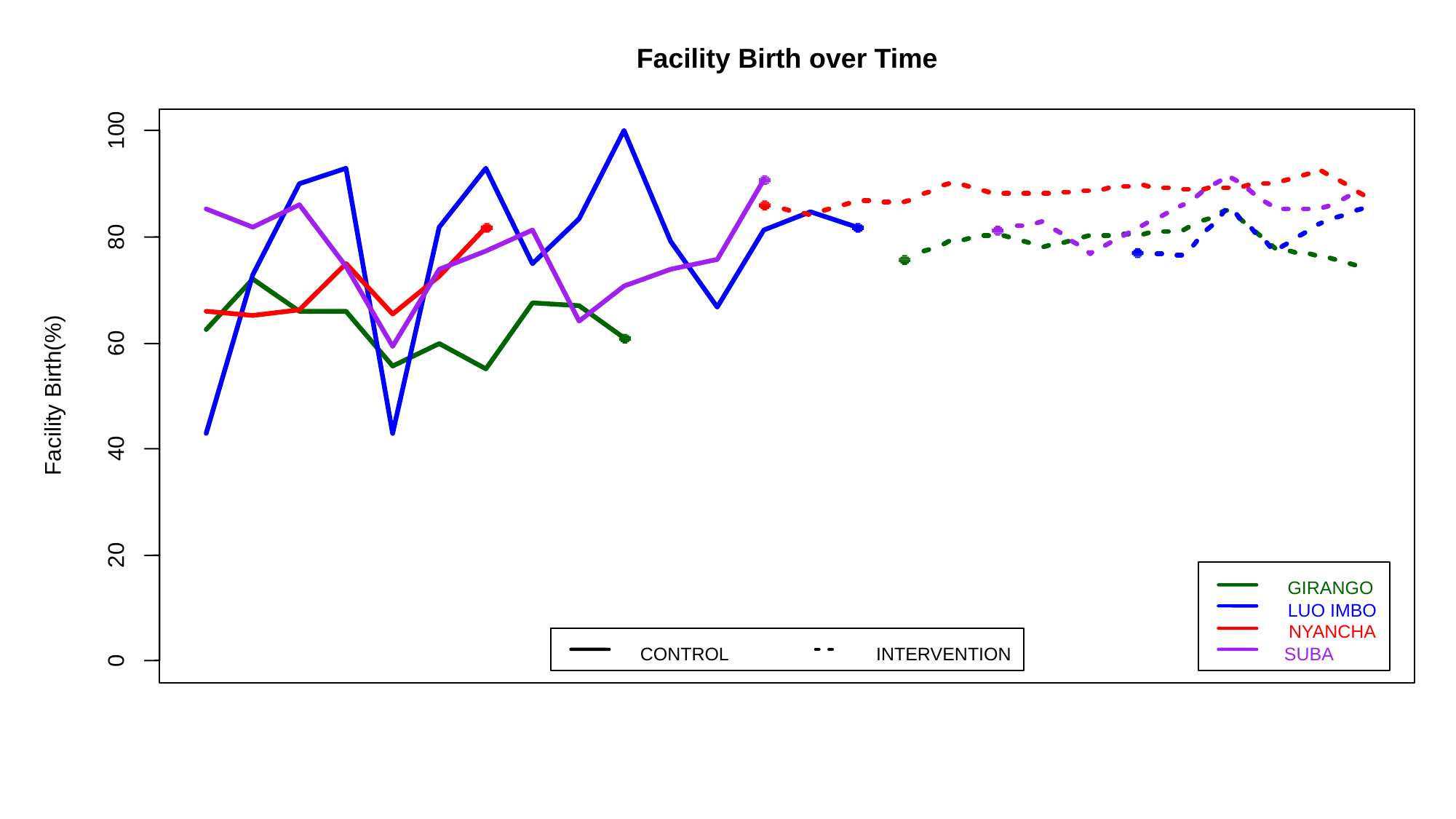

Facility Birth over Time
100
80
60
Facility Birth(%)
40
20
GIRANGO
LUO IMBO
NYANCHA
CONTROL
INTERVENTION
SUBA
0

## Slide 2
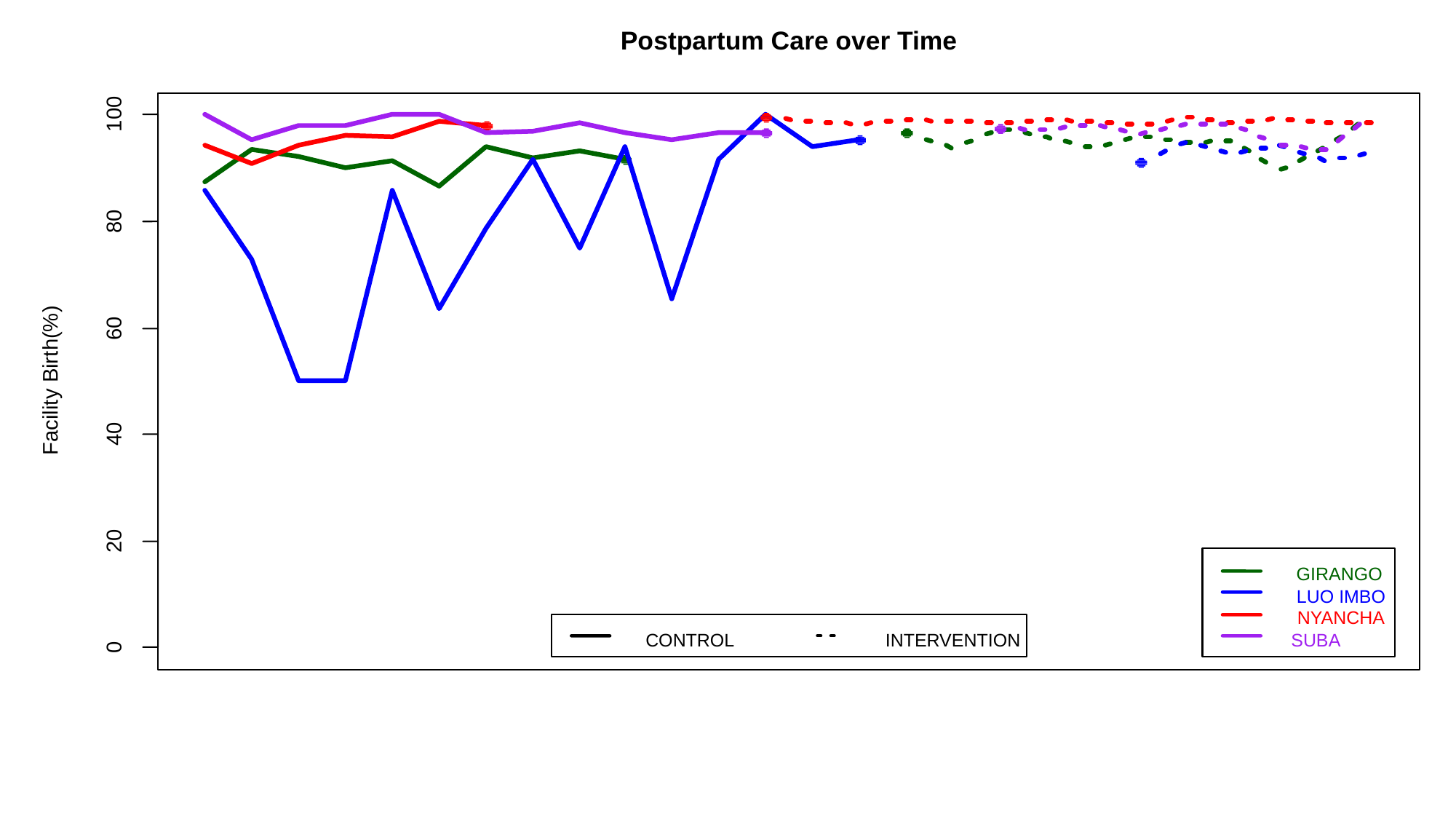

Postpartum Care over Time
100
80
60
Facility Birth(%)
40
20
GIRANGO
LUO IMBO
NYANCHA
CONTROL
INTERVENTION
SUBA
0

## Slide 3
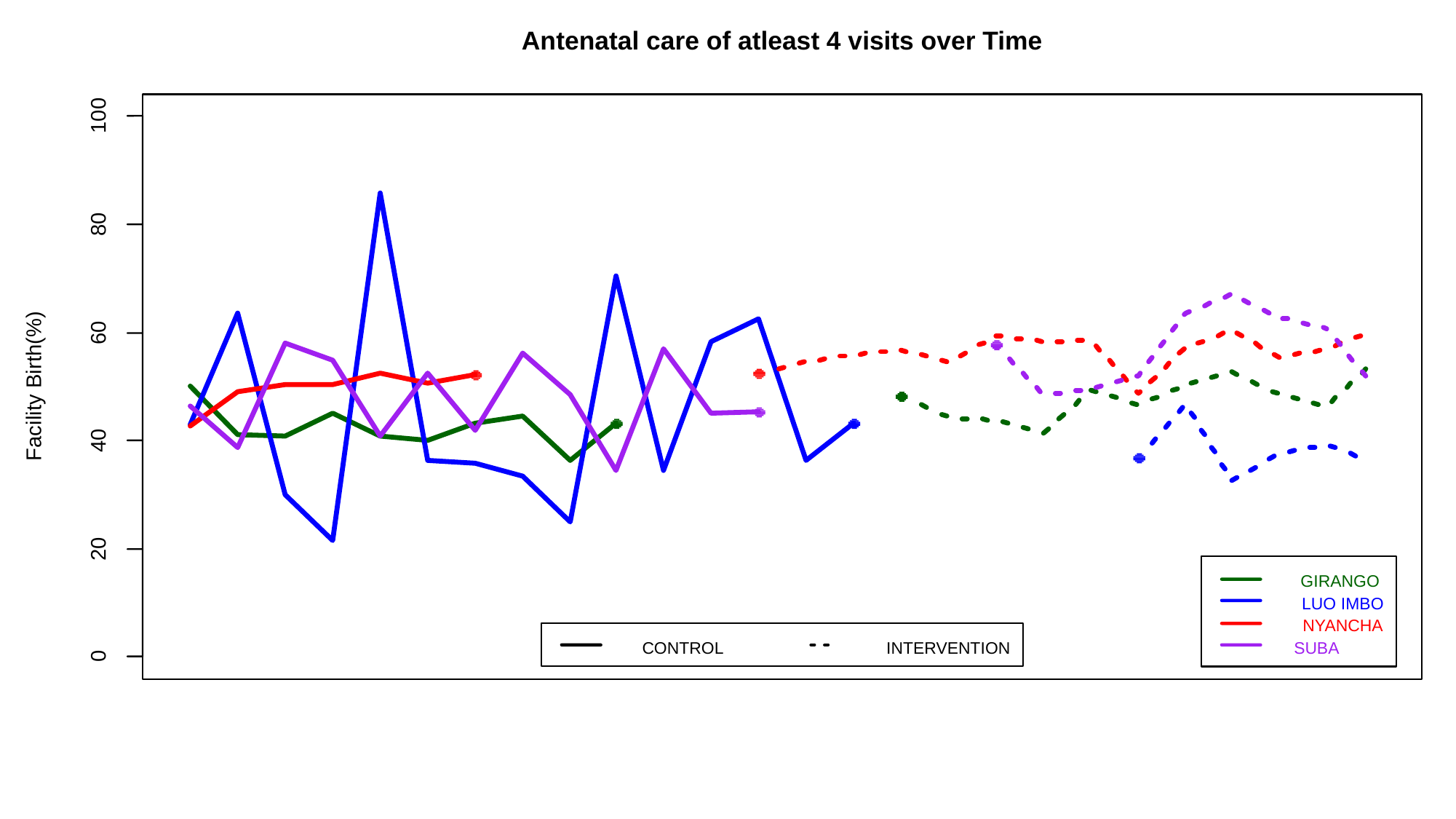

Antenatal care of atleast 4 visits over Time
100
80
60
Facility Birth(%)
40
20
GIRANGO
LUO IMBO
NYANCHA
CONTROL
INTERVENTION
SUBA
0

## Slide 4
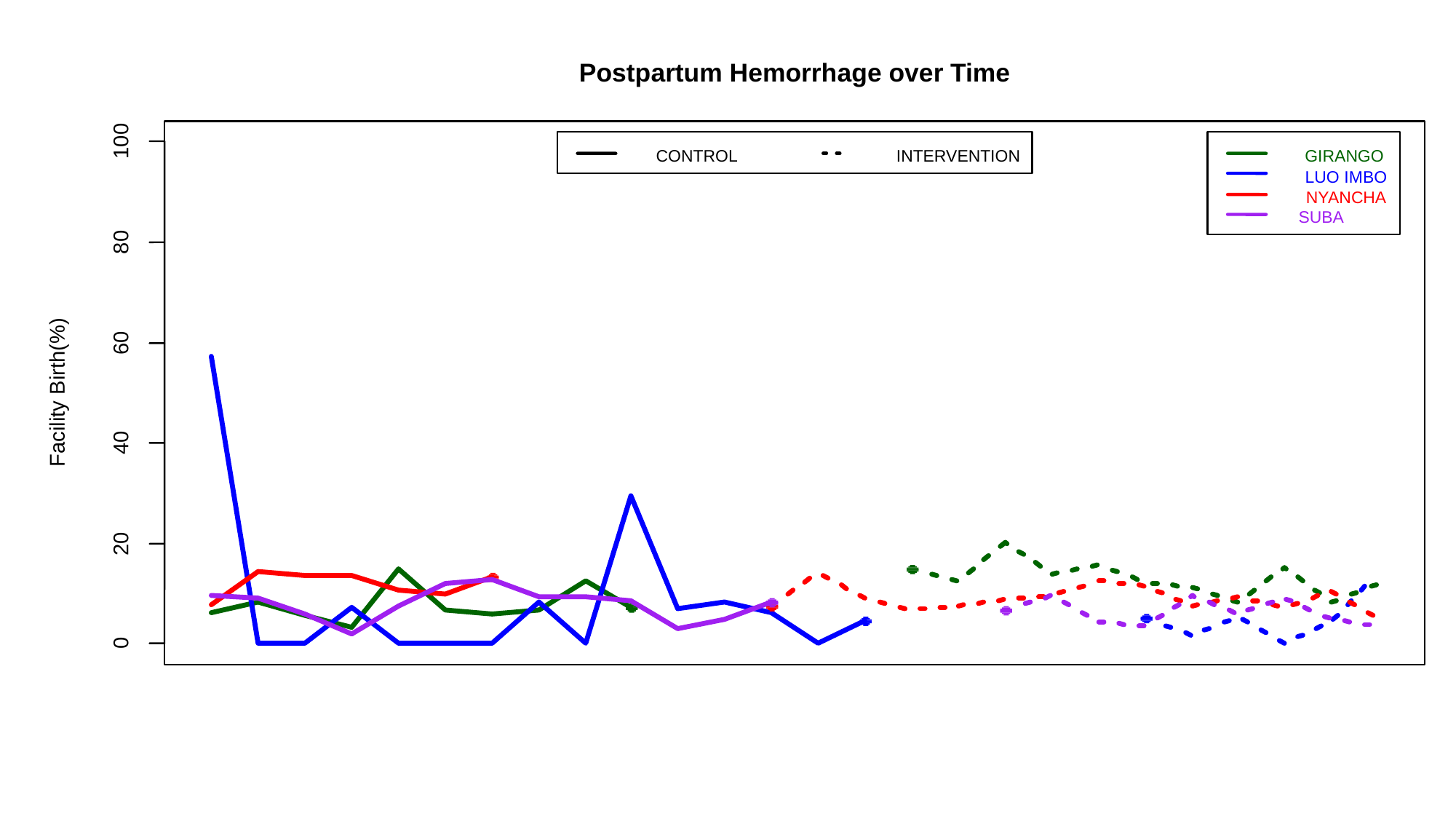

Postpartum Hemorrhage over Time
100
CONTROL
INTERVENTION
GIRANGO
LUO IMBO
NYANCHA
SUBA
80
60
Facility Birth(%)
40
20
0
