# Supplementary material for: Improving health care facility birth rates in Rorya District, Tanzania: a multiple baseline trial
Source: BMC Pregnancy Childbirth. 2022 Jan 27;22:74. doi: 10.1186/s12884-022-04408-5 (PMC8793235; doi:10.1186/s12884-022-04408-5)
Supplement: Supplementary file 2 — Additional file 2. [file 12884_2022_4408_MOESM2_ESM.docx]

**Supplementary Table 1: Results from the segmented logistic regression analysis of the primary and secondary outcomes exploring differences across divisions in the immediate and gradual effects of the intervention (full interaction model)**

|  | **Odds Ratio** | **Lower CL** | **Upper CL** | **p-value** | **p-value for differences across groups** |
| --- | --- | --- | --- | --- | --- |
| **Facility births (Primary outcome)** |  |  |  |  |  |
| Baseline intercept |  |  |  |  | 0.0270 |
| Girango | 0.55 | 0.32 | 0.95 | 0.032 |  |
| Luo Imbo | 0.91 | 0.38 | 2.15 | 0.8233 |  |
| Nyancha | 0.48 | 0.28 | 0.80 | 0.0052 |  |
| Suba | Reference | - | - | - |  |
| Secular trend (change per month before intervention) |  |  |  |  | 0.0372 |
| Girango | 0.98 | 0.93 | 1.04 | 0.5493 |  |
| Luo Imbo | 1.03 | 0.96 | 1.11 | 0.3623 |  |
| Nyancha | 1.12 | 1.04 | 1.20 | 0.0017 |  |
| Suba | 1.02 | 0.97 | 1.06 | 0.5102 |  |
| Immediate effect (intercept change) |  |  |  |  | 0.0082 |
| Girango | 2.60 | 1.46 | 4.64 | 0.0011 |  |
| Luo Imbo | 0.51 | 0.18 | 1.47 | 0.2124 |  |
| Nyancha | 0.94 | 0.51 | 1.73 | 0.8459 |  |
| Suba | 0.83 | 0.48 | 1.44 | 0.4993 |  |
| Gradual effect (slope change, per month) |  |  |  |  | 0.0465 |
| Girango | 1.01 | 0.95 | 1.09 | 0.7161 |  |
| Luo Imbo | 1.05 | 0.84 | 1.32 | 0.6523 |  |
| Nyancha | 0.92 | 0.86 | 1.00 | 0.0426 |  |
| Suba | 1.08 | 1.00 | 1.18 | 0.0597 |  |
| **Antenatal Care of at least 4 visits** | **OR** | **LCL** | **UCL** | **p-value** | **p-value for differences across groups** |
| Baseline intercept |  |  |  |  | 0.7273 |
| Girango | 0.79 | 0.49 | 1.28 | 0.3389 |  |
| Luo Imbo | 0.73 | 0.35 | 1.49 | 0.3807 |  |
| Nyancha | 0.84 | 0.53 | 1.32 | 0.442 |  |
| Suba | Reference | - | - | - |  |
| Secular trend (change per month before intervention) |  |  |  |  | 0.5020 |
| Girango | 0.99 | 0.94 | 1.04 | 0.6421 |  |
| Luo Imbo | 1.01 | 0.95 | 1.07 | 0.818 |  |
| Nyancha | 1.04 | 0.98 | 1.14 | 0.1798 |  |
| Suba | 0.99 | 0.95 | 1.03 | 0.5901 |  |
| Immediate effect (intercept change) |  |  |  |  | 0.5654 |
| Girango | 1.31 | 0.76 | 2.27 | 0.3273 |  |
| Luo Imbo | 0.83 | 0.36 | 1.93 | 0.671 |  |
| Nyancha | 0.86 | 0.51 | 1.45 | 0.5742 |  |
| Suba | 1.26 | 0.81 | 1.96 | 0.3064 |  |
| Gradual effect (slope change, per month) |  |  |  |  | 0.1991 |
| Girango | 1.04 | 0.97 | 1.11 | 0.2427 |  |
| Luo Imbo | 0.96 | 0.80 | 1.16 | 0.6942 |  |
| Nyancha | 0.97 | 0.91 | 1.03 | 0.3179 |  |
| Suba | 1.06 | 1.00 | 1.13 | 0.0694 |  |
| **Post-partum care** | **OR** | **LCL** | **UCL** | **p-value** | **p-value for differences across groups** |
| Baseline intercept |  |  |  |  | <0.0001 |
| Girango | 0.14 | 0.03 | 0.53 | 0.0043 |  |
| Luo Imbo | 0.02 | 0.00 | 0.06 | <.0001 |  |
| Nyancha | 0.12 | 0.03 | 0.49 | 0.003 |  |
| Suba | Reference | - | - |  |  |
| Secular trend (change per month before intervention) |  |  |  |  | 0.0009 |
| Girango | 1.02 | 0.93 | 1.12 | 0.6759 |  |
| Luo Imbo | 1.18 | 1.09 | 1.28 | <.0001 |  |
| Nyancha | 1.29 | 1.10 | 1.52 | 0.0022 |  |
| Suba | 0.93 | 0.82 | 1.05 | 0.2441 |  |
| Immediate effect (intercept change) |  |  |  |  | 0.1903 |
| Girango | 1.79 | 0.64 | 4.99 | 0.2653 |  |
| Luo Imbo | 0.42 | 0.10 | 1.80 | 0.2444 |  |
| Nyancha | 0.46 | 0.10 | 2.18 | 0.3272 |  |
| Suba | 2.19 | 0.57 | 8.41 | 0.2519 |  |
| Gradual effect (slope change, per month) |  |  |  |  | 0.1707 |
| Girango | 0.94 | 0.83 | 1.06 | 0.326 |  |
| Luo Imbo | 0.87 | 0.62 | 1.22 | 0.4154 |  |
| Nyancha | 0.77 | 0.64 | 0.92 | 0.0047 |  |
| Suba | 1.02 | 0.84 | 1.25 | 0.8117 |  |
| **Postpartum Hemorrhage** | **OR** | **LCL** | **UCL** | **p-value** | **p-value for differences across groups** |
| Baseline intercept |  |  |  |  | 0.3198 |
| Girango | 0.73 | 0.30 | 1.81 | 0.5021 |  |
| Luo Imbo | 1.83 | 0.54 | 6.24 | 0.3342 |  |
| Nyancha | 1.43 | 0.65 | 3.12 | 0.3726 |  |
| Suba | Reference | - | - | - |  |
| Secular trend (change per month before intervention) |  |  |  |  | 0.3717 |
| Girango | 1.04 | 0.95 | 1.15 | 0.3794 |  |
| Luo Imbo | 0.92 | 0.82 | 1.03 | 0.1341 |  |
| Nyancha | 1.01 | 0.92 | 1.12 | 0.769 |  |
| Suba | 0.99 | 0.92 | 1.06 | 0.7383 |  |
| Immediate effect (intercept change) |  |  |  |  | 0.605 |
| Girango | 1.64 | 0.64 | 4.23 | 0.3074 |  |
| Luo Imbo | 0.90 | 0.12 | 6.49 | 0.9132 |  |
| Nyancha | 0.70 | 0.31 | 1.55 | 0.376 |  |
| Suba | 1.04 | 0.44 | 2.45 | 0.9256 |  |
| Gradual effect (slope change, per month) |  |  |  |  | 0.5371 |
| Girango | 0.91 | 0.82 | 1.02 | 0.1031 |  |
| Luo Imbo | 1.22 | 0.79 | 1.89 | 0.3701 |  |
| Nyancha | 0.97 | 0.88 | 1.08 | 0.6099 |  |
| Suba | 0.98 | 0.87 | 1.11 | 0.7793 |  |

*All results were obtained from a segmented logistic regression analysis adjusted for age, marital status, education and parity. Interactions between division and each of the time, intervention and time after intervention variables were included and used to produce the division-specific estimates.
